# Supplementary material for: Crystal Structure of African Swine Fever Virus A179L with the Autophagy Regulator Beclin
Source: Viruses. 2019 Aug 27;11(9):789. doi: 10.3390/v11090789 (PMC6784060; doi:10.3390/v11090789)
Supplement: Supplementary file 1 [file viruses-11-00789-s001.pdf]

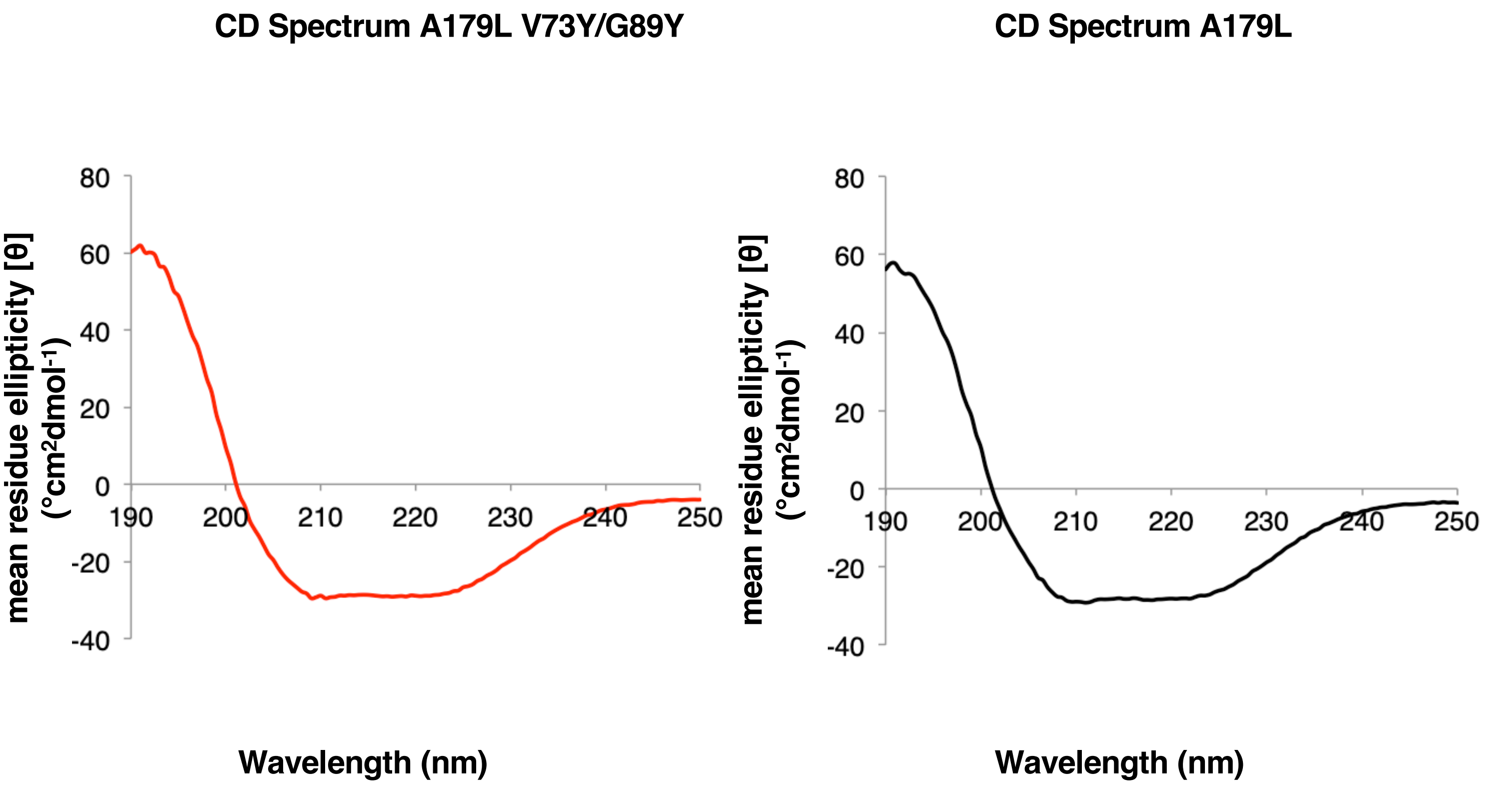

Figure S1 Circular dichroism spectroscopy of wildtype A179L and mutant A179L V73Y/G89Y.

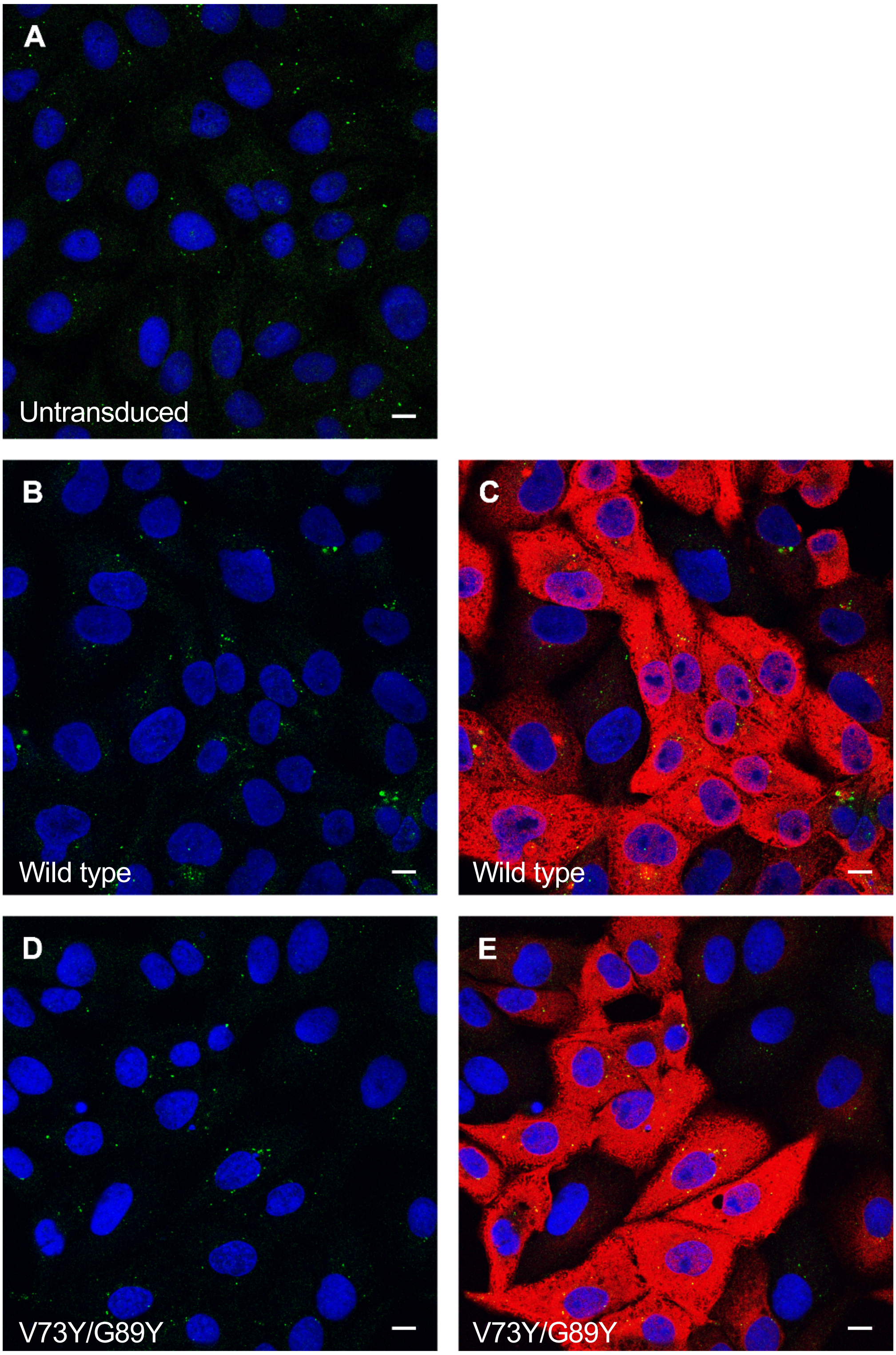

Figure S2 Transduction of Vero cells with either wildtype or A179L V73Y/G89Y does not induce autophagy. Vero cells were transduced with adenoviruses encoding either wildtype A179L (Panels B and C) or A179L V73Y/G89Y (Panels D and E) and were incubated for 24 hours during which cells were incubated in complete Vero cell media for the final 3 hours. Additionally, control cells that had not been transduced were incubated in complete Vero cell media for 3 hours (Panel A). Cells were fixed and permeabilised in methanol and then labelled for LC3B shown in green, HA-tagged protein shown in red and nuclei shown in blue. Panels B and D show the same cells as Panels C and E respectively but with the red channel removed to allow for clearer observation of LC3B staining. Scale bars represent 10 μM.

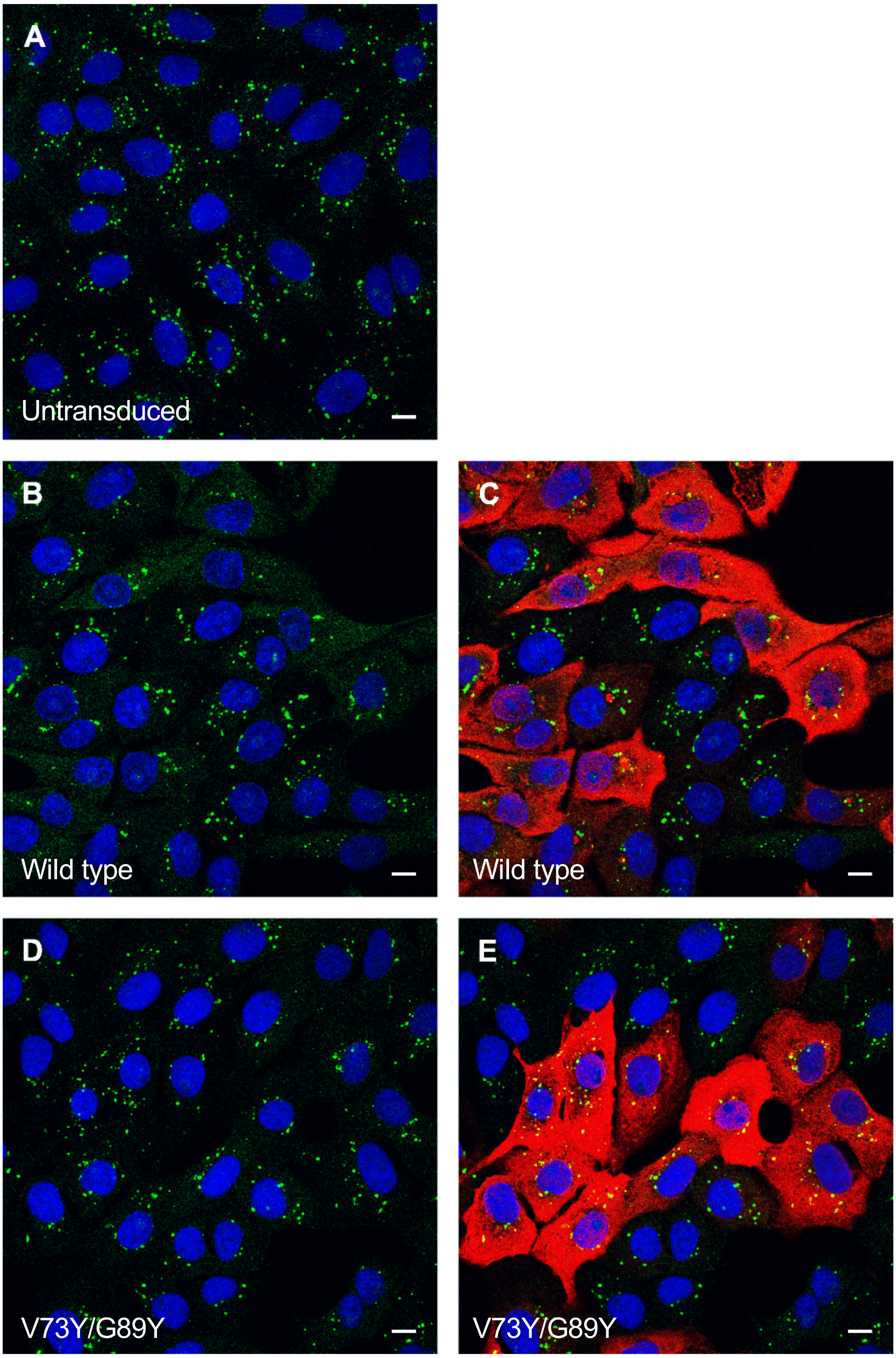

Figure S3 Wild type A179L suppresses the formation of starvation-induced autophagosomes but A179L V73Y/G89Y does not. Vero cells were transduced with adenoviruses encoding either wildtype A179L (Panels B and C) or A179L V73Y/G89Y (Panels D and E) and were incubated for 24 hours during which cells were starved in EBSS media for the final 3 hours. Additionally, control cells that had not been transduced were incubated in EBSS media for 3 hours (Panel A). Cells were fixed and permeabilised in methanol and then labelled for LC3B shown in green, HA-tagged protein shown in red and nuclei shown in blue. Panels B and D show the same cells as Panels C and E respectively but with the red channel removed to allow for clearer observation of LC3B staining. Scale bars represent 10 μM.
